# The Use of the Overmolding Technique for the Preparation of Basalt Fiber (BF)-Based Composite, the Comparative Study of Poly(ethylene terephthalate)/Polycarbonate—PET/PC and Poly(butylene terephthalate)—PBT/PC Blends

**DOI:** 10.3390/polym18010054

**Published:** 2025-12-24

**Authors:** Jacek Andrzejewski, Wiktoria Gosławska, Michalina Salamaga, Weronika Zgoła, Mateusz Barczewski

**Affiliations:** 1 Institute of Material Technology, Poznan University of Technology, Piotrowo 3 Str., 61-138 Poznan, Poland; mateusz.barczewski@put.poznan.pl; 2 Faculty of Materials Engineering and Technical Physics, Poznan University of Technology, Piotrowo 3 Str., 60-965 Poznan, Poland; wiktoria.goslawska@student.put.poznan.pl; 3 Faculty of Mechanical Engineering, Poznan University of Technology, Piotrowo 3 Str., 60-965 Poznan, Poland; michalina.salamaga@gmail.com (M.S.); werko147@wp.pl (W.Z.)

**Keywords:** polymer blends, thermoplastic polyesters, basalt fiber, injection molding, mechanical performance, heat resistance, hybrid materials

## Abstract

The presented study is focused on the evaluation of the mechanical and heat resistance performance of the polyester-based injection-molded components. For comparative purposes, we used a poly(ethylene terephthalate)/polycarbonate blend (PET/PC) and a poly(butylene terephthalate)/polycarbonate (PBT/PC) mixture, where both types of polymer blends were used as a matrix for different types of basalt fiber (BF)-reinforced composites. The investigated molding procedure consists of injection overmolding of the composite prepreg (insert). During the technological procedure, various material configurations were used, including overmolding with both unmodified blends and a composition with additional short basalt fibers. The results confirmed that the best balance of properties was obtained for complex parts reinforced with short BF and overmolded insert, where the tensile modulus can reach 8 GPa, while the impact strength was more than 30 kJ/m^2^. The results of comparative tests indicate a significantly higher strength of overmolding joints for PET/PC-based materials. The relatively low heat deflection temp. (HDT) of around 70 °C after the injection molding procedure can be successfully improved by the annealing treatment, where the HDT can reach around 120 °C. The structural tests revealed that, besides some differences in crystallinity between the PET- and PBT-based blends, the thermomechanical performance of the manufactured composites is almost similar. It is worth pointing out the fundamental differences in the miscibility of the investigated blend systems, where for the PBT/PC mixture structural tests confirm the miscibility of polymer phases, while PET/PC particles are immiscible.

## 1. Introduction

For decades, the production of lightweight structures has been one of the main reasons for using polymer composites. This trend has become widespread in the mass production of automotive and aerospace components, sports equipment and many other industries where operating conditions allow for the replacement of metal components with various types of polymer-based composites. So far, the most effective way to reduce the weight of the final products has been the use of thin-walled composite structures reinforced with carbon fibers or, more commonly, glass fibers [1,2,3,4]. Traditionally, the base for these types of materials was epoxy and polyester resins. Due to the difficulties with recycling, these types of materials are treated as hazardous waste and are most often landfilled; an example being the process of recycling wind turbine blades [5,6,7], which is unprofitable from an economic point of view. In the case of carbon fiber composites, the recycling process makes much more sense due to the possibility of reusing expensive carbon fibers [8,9,10], but in most cases the fiber recovery process involves the pyrolytic removal of the polymer matrix, which consequently eliminates the possibility of reusing the polymer material [11,12,13]. Numerous attempts are currently being made to replace traditional chemically cured resins with recyclable materials. In the case of traditional liquid resin processing techniques, such as resin transfer molding or resin infusion, the use of vitrimers seems promising [14,15,16,17]. However, at the current stage of industrial development, this does not yet have much of a prospect for widespread use. It is possible to more widely use solutions based on thermoplastic polymers but, due to the different technologies of their shaping, this sometimes requires significant interference in the technological lines [18,19,20,21].

In the case of standard thermoplastic processing techniques (extrusion, injection molding), the composite reinforcement is in the form of short fibers with a maximum length of 2–3 mm. A certain exception is the LFT technique [22,23,24], where the fiber length can reach up to 20 mm; however, from the point of view of the effectiveness of mechanical interactions, materials of this type cannot compete with traditional laminates reinforced with continuous fibers [25,26]. In contrast, the methods of manufacturing laminates based on thermoplastic polymers are always associated with the need to use pressing techniques, where the fibrous reinforcement in the form of a mat or fabric is combined with the matrix at the stage of plasticizing the polymer film or fibers [21,27,28,29]. Unfortunately, in this type of process, the possibilities of manufacturing complex shapes are limited to flat, thin-walled products, which is not in competition for the injection molding method. The overmolding technique of composite inserts discussed in the article is therefore an example of combining the advantages of using long fiber-reinforced laminates with the efficiency and flexibility of the injection molding method. Previous research and industrial implementations of this concept included various material and technological variants, but the key stage of the process was always placing the composite prepreg inside the injection mold and then overmolding it with the molten polymer material supplied to the mold [30,31,32,33,34]. The procedure is analogous to the overmolding of metal inserts but several important details distinguish the two processes. In the case of metal inserts, these elements are usually small, for example, threaded inserts [35,36,37,38]. Under the conditions of the injection molding process (200–300 °C) these materials do not melt, necessitating the use of form-fit joints filled with polymer during the injection stage. Composite prepregs are usually in the shape of flat inserts adhering to a large surface of the injection mold. In this case, a permanent connection is obtained by diffusion connection of the insert with the over-injected material, which takes place during filling of the mold cavity. While the issue of the strength of this type of part for metal inserts depends on the product design, for composite prepregs it is the result of the thermal parameters of the process and the materials structure/composition [31,39,40,41].

In the case of the currently discussed research, a PET/PC and PBT/PC polymer mixture system was used, which is a novelty in the context of previous work in the field of prepreg overmolding technology [33,42]. In fact, the literature study revealed that the research topic of PC/polyester-based blends modification and/or manufacturing is relatively not often studied, which has resulted in a limited number of publications on this topic. Unlike studies on PC/ABS blends, which are common in the literature due to their widespread industrial use [43,44,45,46,47], the studies on PC-based blends with composite additives are less common; however, there are some examples regarding the use of micro/nano fillers [48,49,50,51,52]. The concept of the work, taken from previous publications on this topic, assumes that composite prepregs placed inside the injection mold will not be subjected to additional plasticization before the overmolding process, which shortens the entire procedure by one stage [34,53]. The PC/polyester types of polymer blends, apart from being the most commonly used in industry PC/ABS systems [44,45,47,54], are examples of technical blends intended for demanding engineering applications [55,56,57,58,59]. The advantages of PC in the form of favorable impact strength and thermal resistance (high glass transition temperature), combined with chemical resistance and excellent processability (low viscosity) of PET and PBT components, result in materials of this type being used in many industries, despite their high price. The previously conducted studies dealt with a concept of PC/PET blend overmolding procedure [33,53,60], where the prepreg insert was made from self-reinforced PET-based material. Selected results indicated the advantage of this solution over unmodified PET, which is the basis for developing the concept of the work in the currently described research. The first step was to replace the srPET prepreg with a composite reinforced with basalt fibers (BF insert), and the next step was to extend the research to blend systems containing PBT, as this material usually has higher crystallinity. The direction of the work was aimed at obtaining the highest possible heat resistance value; hence, the manufacturing methodology included not only the overmolding process but also annealing in order to increase thermomechanical stability [61,62,63,64,65].

The research work carried out within the presented project consists of a multi-stage process of manufacturing composite prepregs, their injection overmolding and thermal treatment (annealing). The overmolded stage was performed using both unmodified blends (PET/PC, PBT/PC), while additionally, the 20% basalt fiber composite was used. The properties of the obtained materials were evaluated using mechanical tests (static tensile, Charpy impact method, interlaminar shear strength test), thermal analysis methods (DSC, DMTA) and heat resistance tests (Vicat, HDT). The study was supplemented with sample appearance photos. Apart from the obvious research objective of describing the characteristics of materials after the overmolding process, the experiments carried out are aimed at evaluating the strength of the joint created during the mold filling stage. Such a preliminary assessment will allow for the selection of a material system with greater potential for further development work.

## 2. Research Methodology

### 2.1. Materials

Three types of polymers were used for the preparation of the polymer blends used during this study. Ultradur B4250 PBT—injection molding grade poly(butylene terephthalate) resin from BASF Engineering Plastics (Ludwigshafen, Germany), with the MVR (melt volume-flow rate) = 25 g/10 min (250 °C; 2.16 kg). Ramapet N1 PET—bottle grade poly(ethylene terephthalate) resin from Indorama Ventures (Wloclawel, Poland), with the IV (intrinsic viscosity) = 0.78–0.82 dL/g. Makrolon 2205 PC—general purpose polycarbonate resin from Covestro AG (Kaiserslautern, Germany), with the MVR = 34 g/10 min (300 °C; 1.2 kg). All materials were supplied in the form of pellets. Before the melt mixing procedure the pellets were dried for 12 h at 80 °C (cabinet drier).

Short basalt fibers in the form of chopped strand were purchased from Kamenny Vek Co. (Dubna, Russia); the fiber type BCS13-3.2 mm(1/4″)-KV-18 is µm in diameter and 3.2 mm long. The basalt fabric was purchased from the same producer (Kamenny Vek, Dybna, Russia); the BT11/1 twill fabric type was used as received, and the fabric weight was 210 g/m^2^. In order to avoid surface moisture the chopped fibers and fabrics were stored in the cabinet dryer (2 h, 100 °C).

### 2.2. Sample Preparation

The melt mixing of the pure blends and composites was performed using a co-rotating twin screw extruder, type ZAMAK EH.16D (Zamak Mercator, Krakow, Poland). For all materials, the temperature profile was set as follows: 250 (1st zone)–260–260–265–265–270–270–260–260 (die). The extruder screw speed was set to 100 rpm. The extrudate was cooled and pelletized. Before the next step of processing, pellets were dried (12 h, 80 °C). The list of prepared samples together with the formulations are collected in Table 1.

**Figure 1:**
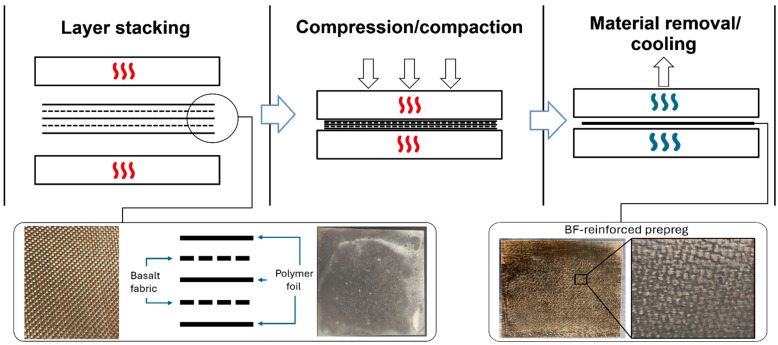
The scheme presents the composite manufacturing procedure.

Prepreg composites were prepared using the film-stacking method. For this purpose, polymer foils were prepared in the first stage. A small amount of pellets (≈15 g) was placed between two PTFE sheets. The material was then inserted between the heated hydraulic press tables. The press type we used was LABManual 300 (Fontijne Presses, Delft, The Netherlands). The process temperature was set to 270 °C, while the maximum pressure was 200 bar. After obtaining a 0.2 mm thick film, the material was cooled by placing it outside the heated plates; additional cooling was not necessary. The foils obtained during the compression process were cut to a 200 mm × 200 mm size and then used for the production of composites. Composite prepregs were made in two versions. Thin with a thickness of about 0.7 mm made by pressing 2 layers of fabric and 3 foils. The production of composites was similar to the method of film preparation, but in the final stage the composites were placed between the cold plates of the second press to cool down. The temperature of the heated plates was again 270 °C, the maximum applied pressure 100 bar, while the maximum load time was 10 min. The composite prepreg preparation procedure is presented in the scheme in Figure 1.

**Figure 2:**
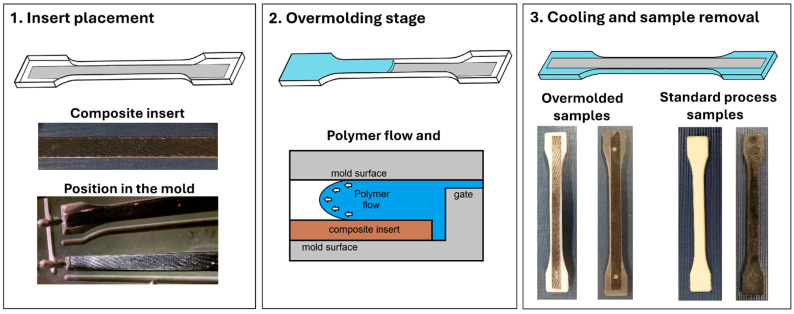
The scheme presents the overmolding stage of the preformed procedure.

The injection molding tests were carried out using the Engel e-mac 50 injection molding machine (Engel GmbH, Schwertberg, Austria). The maximum injection molding temperature of 280 °C was used for all types of materials; the injection pressure was 1150 bar, the holding pressure was 550 bar and the holding pressure time was 10 s. The samples were cooled for 45 s, and the mold temperature was 80 °C. During the overmolding tests, the composite prepregs were placed inside the mold cavity. In order to compensate for the difference in the volume of the mold, the volume of a single injection was reduced from 38 cm^3^ to 37 cm^3^, which helps to avoid flashing errors. Before each injection cycle, the surface of the prepregs was cleaned with acetone, which avoided problems with adhesion to the composite surface. The short description of the overmolding process stages is presented in Figure 2.

### 2.3. Characterization

The mechanical performance of the prepared composites was evaluated using the static tensile measurements and notched Charpy impact tests. Static tensile measurements were conducted using an Instron 4481 machine. The injection-molded samples were prepared according to the ISO 527 standard [66], where a 1A type of specimen was molded. The tensile test speed was 5 mm/min, and the gauge length was 50 mm. The prepreg laminates were tested using the same conditions.

Impact strength measurements were conducted using the notched Charpy method. Tests were carried out according to the ISO 179 standard [67] using the HIT25 pendulum machine (Zwick/Roell GmbH, Ulm, Germany). The hammer energy was 5 J. After the molding stage samples were notched, and the notch depth was 2 mm. The impact force was recorded during the measurements. For injection-molded samples the specimen cross-section was 4 mm × 10 mm, while for the prepreg laminates it was 1 mm × 10 mm.

For all samples the DMTA (dynamic mechanical thermal analysis) measurements were carried out in the temperature range from 30 to 180 °C, with a heating rate of 2 °C/min. The tests were conducted in torsion mode using an MCR301 rotational rheometer equipped with a heating chamber (Anton Paar GmbH, Graz, Austria). The value of the deformation amplitude was 0.01% while the frequency was 1 Hz.

Differential scanning calorimetry (DSC) thermal analysis was carried out on small-sized samples cut from the material samples. A sample of 5–10 mg was placed in an aluminum crucible and secured with a lid. The material prepared in this way was placed in the measuring device of the apparatus. The test was carried out in the temperature range 20–300 °C, with a heating/cooling rate of 10 °C/min. Measurements were carried out under a nitrogen atmosphere; the flow intensity in the chamber was 10 mL/min. DSC F1 204 Phoenix (from Netzsch GmBH, Selb, Germany) was used in the research. The analysis of thermal transformations was carried out on the basis of the first heating and cooling stage thermograms.

For fiber-reinforced materials, visual observation of the structure may be informative. Unfortunately, due to difficulties in obtaining the correct fracture for the laminate, even with samples conducted with liquid nitrogen, we were unable to prepare a suitable sample; hence, for the discussed research, we remained with the optical analysis of the fracture of the samples, an analysis which was conducted using the optical microscopy. Pictures were taken with an Opta-Tech MB200s optical microscope (Opta-Tech, Warsa, Poland) equipped with a Meiji Techno HD2600T camera (Meiji Techno America, Campbell, CA, USA).

## 3. Results and Discussion

### 3.1. Mechanical Performance—Static Measurements, Impact Tests and Interlaminar Shear Strength Analysis

**Figure 3:**
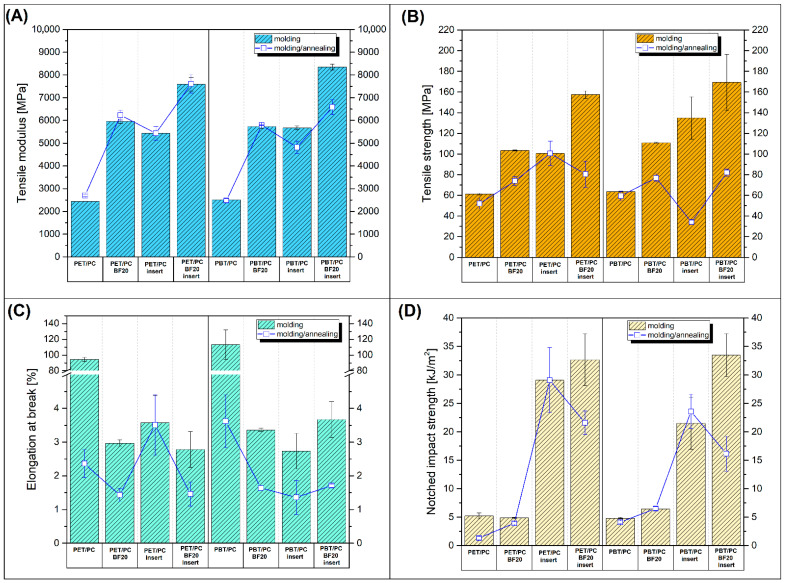
The result of the mechanical performance measurements, static tensile and Charpy tests: (**A**) tensile modulus; (**B**) tensile strength; (**C**) elongation at break; (**D**) Charpy impact strength. The results reflect the properties of molded (columns) and annealed samples (point-line).

The results of mechanical measurements cover a wide range of prepared materials; therefore, the charts in the figures present the most important results only for selected samples, since the overall results have been included in the Table S1 (Supplementary Information Section). The Figure 3A,B. presents the tensile modulus and strength results comparison for PET/PC- and PBT/PC-based materials, while plots from Figure 3C,D reveal the results of elongation at break and Charpy impact strength. The appearance of the tensile test samples after fracture is presented in Figure S1 (Supplementary Information Section).

Even a cursory results analysis indicates a significant difference in the material properties between the sample series; however, taking into account that the graph presents results for standard injection-molded samples and insert-reinforced ones, the differences in values are justified. Initial values obtained for pure PET/PC and PBT/PC blends can therefore be considered a reference, and the analysis primarily aims to assess the effectiveness of various forms of basalt reinforcement. Basalt fiber was used in the form of dispersed short fibers (BF), overmolded prepreg (insert) or in both forms (BF/insert). An upward trend is observed for all materials with the addition of basalt fibers. The use of double modification (BF/insert) gives the best results. For example, the tensile modulus of the PET/PC-BF20/insert sample increased to almost 7600 MPa from the initial 2442 MPa for the PET/PC blend. For the same samples, the tensile strength increased from 61 MPa to 157 MPa. It is worth noting that the reference blend samples, PET/PC and PBT/PC, have very similar properties, as do the varieties reinforced with short fibers (BF20). Some minor differences in values relate to the tensile strength/modulus of prepreg samples, where the PET/PC-prepreg has slightly higher strength than the PBT/PC-prepreg sample (see Table S1). However, considering the scale of this difference, where the strength-to-modulus ratio for PET/PC-prepreg and PBT/PC-prepreg are 366/345 MPa and 25/23 GPa, respectively, it can be assumed that the prepreg properties are similar regardless of the used matrix blend. Interestingly, the tensile strength/modulus for prepreg-reinforced specimens are blends with the addition of short BF and are usually very close in value, suggesting the same reinforcing efficiency. It is undeniable that for the prepared materials, the differences in basic mechanical parameters (modulus/strength) caused by the annealing process are not as dramatic as in studies conducted on materials with a homogeneous matrix structure. A popular example is the numerous studies on the annealing procedure for PLA-based materials, where the modulus increase is sometimes significant [64,68,69,70]. The likely reason for the lack of significant changes in the studies discussed is the significant share of the amorphous PC phase (50%). Given the lower density of the PC phase compared to polyesters (1.2 g/cm^3^ vs. 1.3 g/cm^3^), the volumetric ratio favors PC. Any changes occurring within the polyester phase (PET/PBT) will have a minor impact on the main strength parameters, as demonstrated by this study.

In the case of the analysis of the elongation at break value, the measurement results clearly show that for all reinforced samples there was a considerable decrease in the elongation at break values compared to unmodified blends. The initial values for the reference materials, PET/PC = 95% and PBT/PC = 113%, were reduced to below 5% for all composite samples. Interestingly, the elongation values for all samples manufactured by the overmolding method are close to those obtained for the prepreg laminates, indicating a certain correlation in behavior. Regardless of the obtainable elongation values of the injection-molded part, the whole sample is fractured when the prepreg structure delaminates from the molded part. This is strongly in line with the results of our previous research, where polyester-based materials were reinforced with prepregs made from self-reinforced composites (srPET) [42,53]. Unlike many other types of laminated materials, like multicomponent films, where the breakage of a single layer of the materials does not lead to complete fracture, for the investigated types of composites the crack propagation transfers from the prepreg part to the injection-molded section of the specimen. This kind of behavior can be considered unfavorable; however, the continuous crack propagation confirmed the high strength of the interface between the insert and the molded part, which was the main goal during the development of the presented manufacturing concept.

The analysis of the Charpy impact test results seems to reveal the most important features of the investigated composites, since the addition of the prepreg in the sample structure determines a significant improvement in impact strength. Due to the relatively similar impact strength of the reference blends and BF modified samples, the main reason for the increase in impact strength is the use of the long fiber reinforcement in the form of inserts. The best results were recorded for PBT/PC-BF20/insert and PET/PC-BF20/insert composites, 33.5 kJ/cm^2^ and 32.7 kJ/cm^2^, respectively. Compared to the 5 kJ/cm^2^ recorded for unmodified blends, this represents a significant improvement. Slightly lower impact strength improvement was recorded for samples overmolded with an unmodified blend; however, the recorded impact strength values are still several times higher than for reference samples. Although the impact strength of many overmolded samples increased several times, that of the prepregs was not as high as expected. The highest result of 65 kJ/cm^2^ was recorded for PET/PC-prepreg samples, while for PBT/PC-prepreg it was 43.7 kJ/cm^2^. A relatively low result is caused by the methodology of measurements using the Charpy method; due to the use of relatively thin laminates (≈1 mm) the sample rotates during the impact which hinders its frontal fracture. Therefore, the impact strength obtained during the measurements is significantly underestimated. Due to the larger thickness of the injection-molded specimens (4 mm), their ability to rotate during the test is limited, which means that for overmolded materials the structure of the insert can absorb a significantly higher portion of energy, which translates into high impact strength.

Tensile and impact tests were simultaneously conducted on specimens subjected to the annealing procedure, and the results are presented in the same Figure 3 as a scatter plot. In the case of tensile modulus measurement, where it was expected to obtain higher crystallinity of the polyester phase and thus increased stiffness, the results were unexpectedly worse than those of unannealed (molded) samples. Interestingly, for annealed PET/PC-based materials, most of the results are strongly in line with the values recorded before heat treatment, while the measurements conducted for PBT/PC-based materials revealed visible modulus deterioration for overmolded insert specimens. Even a more pronounced reduction in properties is evident in the tensile strength results, where most values after annealing are lower. The most pronounced decrease in tensile strength was observed in overmolded insert materials. This fact suggests that, for materials reinforced with the laminate prepreg, in addition to the normal decrease in strength associated with a higher level of crystallinity, other factors turn out to be decisive, probably related to stress changes at the prepreg/molded part interphase. The lower interphase strength is also confirmed by the delamination observed in the samples subjected to annealing. The analysis of the elongation at break values for annealed materials reveals a large drop in strain for unmodified blends. For the PBT/PC blend, the reference strain of 113% was reduced to 2.4%, while the difference for the PET/PC-based samples was respectively 95% and 3.5%. These unfavorable changes are obviously caused by the increase in the crystalline phase content of both polyesters (PET/PBT).

**Figure 4:**
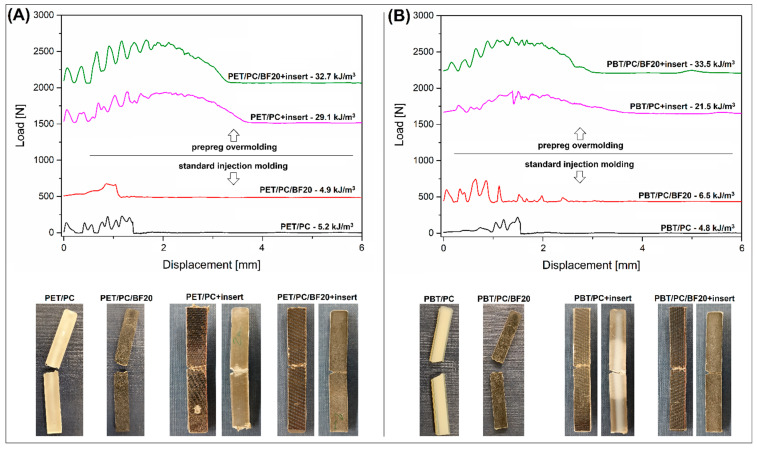
The load-displacement plots recorded during the Charpy measurements: (**A**) for PET/PC-based samples; (**B**) PBT/PC-based materials. The photographs reveal the appearance of the tested specimen after the fracture.

The curves shown in Figure 4 present the process of sample deformation during the Charpy impact test. The load/displacement plot was recorded with the use of a high-speed force transducer located at the point of impact on the pendulum hammer. The graphs show a comparison of the fracture process for a sample based on PET/PC and PBT/PC blends. The appearance of the curve was posted together with the impact strength value obtained for a particular type of composite. The lowest impact strength was obtained for reference blend samples and those reinforced with short BF. For both PET/PC- and PBT/PC-based samples, the impact strength was approximately 5 kJ/m^2^, a characteristic value for this type of material [55,56,71]. Although the impact strength of the PC component is twice as high, the low impact strengths of PBT and PET determine the low impact strength of the blends. The higher energy consumption is confirmed by the appearance of a curve for the samples obtained in the overmolding process. The area under the force plot is several times greater than for samples obtained by the traditional injection molding technique, which is due to both the higher maximum force value and the longer deformation of the sample. This type of fracturing/cracking process confirms the high adhesion of the prepreg structure and the molded material, which was one of the objectives of the research.

**Figure 5:**
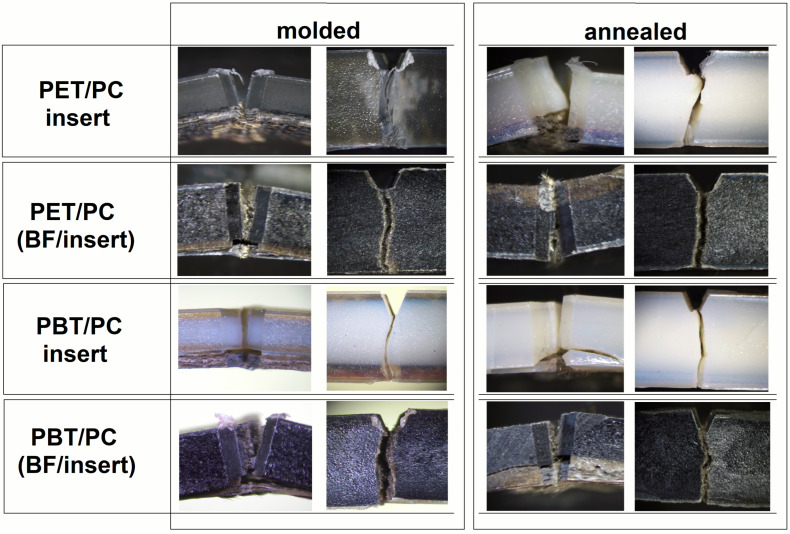
The enlarged view of the notch area of the overmolded samples after the Charpy impact testing. The compilation includes the molded and annealing samples.

A deeper analysis of the impact specimen cracking mechanism is presented in Figure 5, where the notch area for the overmolded samples was inspected by the light microscopy method. A clear tendency that applies to all analyzed fractures is the complete fracture of the overmolded part of the specimen, while in none of the studied cases was the composite insert completely fractured. Analysis of the fracture point of the overmolding part reveals a fundamental difference between the unreinforced materials and those with the addition of BF filler; where, for loaded materials, the basalt fibers are clearly visible in the space between the broken parts of the sample. The visible structure indicates the intensity of the short fiber pull-out mechanism, which, in the case of the investigated materials, indicates the occurrence of relatively low adhesion at the matrix-filler interface; however, this is not a negative feature in the context of impact properties, as confirmed by numerous studies [72,73].

Taking into account the conducted visual analysis, as well as previous results for materials reinforced with BF chopped fibers [74,75,76], the initial fiber length has no significant effect on the obtained properties. For selected works we used 1/4 in. (6.4 mm) or 1/8 in. (3.2 mm) in. BF. Since for most of the research carried out chopped fibers are subjected to compounding on twin-screw machines, the length reduction is so significant that, statistically, the fiber length usually falls within the range of 0.2–0.3 mm. Even for LFT materials, the final fiber size does not exceed 0.8 mm [77,78]. In this study, we did not expect to obtain significantly different results from structural studies of the overmolded part, hence the fracture structure was not analyzed. Of course, the measurement results for the composite prepreg indicate significantly higher impact strength. However, considering previous publications and similar phenomena occurring in glass fiber-based materials, measuring such phenomena would not contribute any new knowledge to the topic [79]. From the perspective of possible applications, however, it would be interesting to conduct a broader characterization of the properties of the prepregs themselves based on PET/PC and PBT/PC blends.

**Figure 6:**
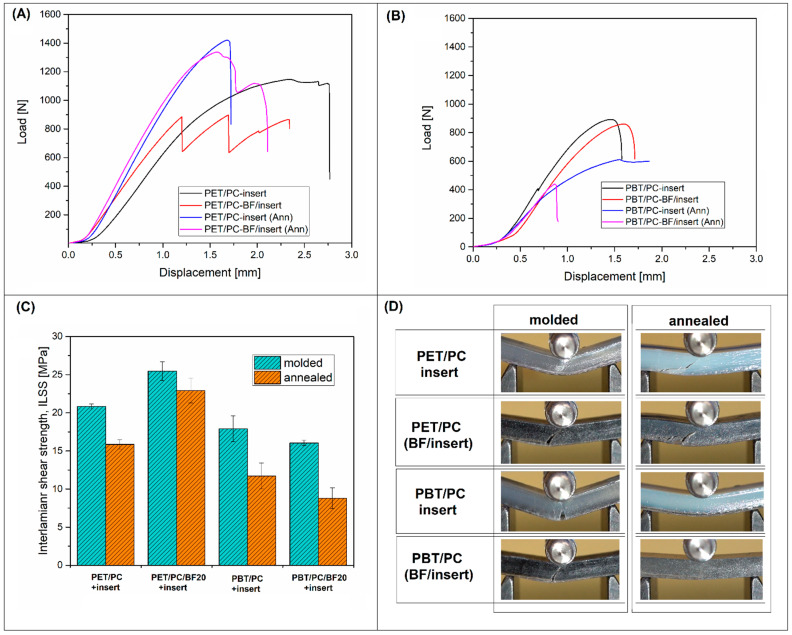
The results of the interlaminar shear strength measurements. The load/displacement plots for (**A**) PET/PC materials and (**B**) PBT/PC samples; (**C**) ILSS calculation results; (**D**) appearance of the specimens during the fracture.

The mechanical performance analysis was supplemented with the interlaminar shear strength tests (ILSS), where the main purpose of these measurements was to evaluate the strength of the connection between the prepreg surface and the overmolded material (see Figure 6). The results in the form of load-displacement plots, ILSS values and sample appearance are collected in Figure 6A,B. For both molded and annealed samples, it is clear that the highest ILSS was obtained for PET-based materials. The plot analysis revealed that not only the maximum load but also the maximum strain range was visibly higher for PET/PC specimens. The sample fracture inspection revealed that the delamination occurred at the prepreg-overmolded side interface, which confirmed that the recorded values are correlated with the interlaminar shear strength. It also confirmed that the higher ILSS values for PET/PC materials are correlated with the effectiveness of diffusivity phenomena during the overmolding process. The possible reason for that might be correlated with the crystallinity level of the semicrystalline component (PET/PBT). According to the thermal analysis results (see Section 3.3), the PBT/PC blend system is miscible. This means that the mobility of the amorphous phase was strongly limited, which reduces the diffusion of macromolecules at the overmolding stage. Additionally, the crystallization kinetics for the PBT phase are much faster than for PET, which reduces the time of possible surface interactions. In contrast, the PET/PC immiscible blend showed slightly lower dynamics of the crystal structure formation process, which causes the diffusion of macromolecules to occur in more favorable conditions. The annealing process leads to a reduction in the ILSS value, which is essentially a consequence of the limited level of maximum deformation of the overmolded sample.

**Figure 7:**
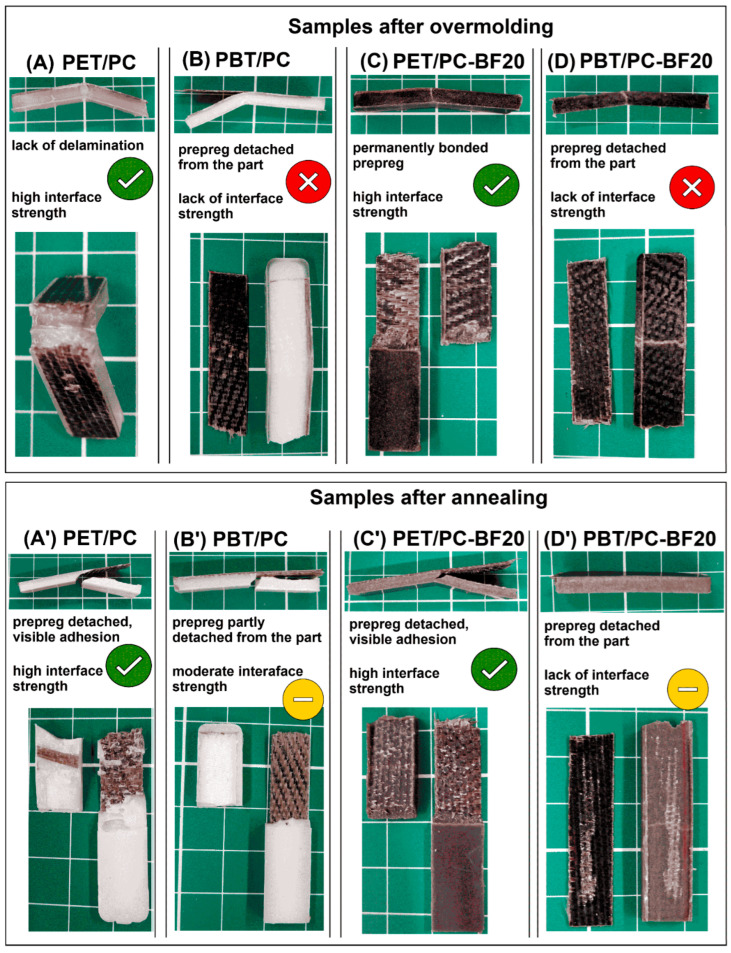
The ILSS test sample appearance after the performed test: (**A**,**A′**) PET/PC; (**B**,**B′**) PBT/PC; (**C**,**C′**) PET/PC-BF20; (**D**,**D′**) PBT/PC-BF20. Samples’ visible differences at the interface surface are correlated with the interface strength results.

Since a particular type of polymer blend was not used for the prepreg overmolding, the obtained ILSS test results cannot be directly compared to other research outcomes; however, some approximations can be made for other types of composites with a similar curing procedure. The closest example of that is the mentioned overmolding of the PA6/carbon fiber tow with an ILSS range around 20–30 MPa [80]. Interestingly, for overmolded highly filled PEEK/CF materials, the ILSS test results were only slightly higher and ranged around 28–39 MPa [81]. It is worth noting that the interfacial strength is often measured by lap shear measurement or other methods [82,83]; therefore, analyses of the literature often result in very large discrepancies in results. Importantly, from the discussed perspective, the fracture mechanism of the investigated samples was consistent with the ILSS test results, as evidenced by the presence of an interface failure at the joint between the two materials (see Figure 7). For the part prepared with the use of a PET/PC-based matrix the prepreg surface was still strongly connected to the overmolded part even after the ILSS test. For most of the parts, only one part of the bar was detached from the insert surface, while the high roughness of the fractured surface and the torn-out fiber fragments indicated a high degree of adhesion at the insert interface. In contrast, the samples prepared with the use of the PBT/PC blend, the appearance of the samples after the test indicates a different behavior; for a large part of the samples, the entire surface of the insert was easily delaminated during the ILSS test. This indicates a lack of strong adhesion at the investigated interface. At the same time, it suggests that for this group of materials the observed properties do not result from a permanent physical bonding of both components of the molded part but are only the result of their mechanical interlocking, without any visible matrix layer attached to the prepreg.

### 3.2. Thermomechanical Properties—DMTA Analysis and HDT Tests

**Figure 8:**
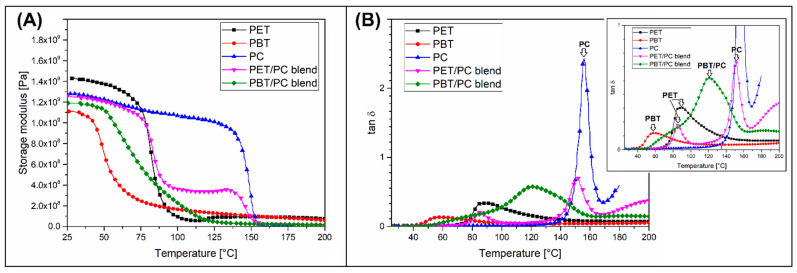
The viscoelastic properties of the reference unreinforced materials: (**A**) storage modulus and (**B**) tan δ plots.

**Figure 9:**
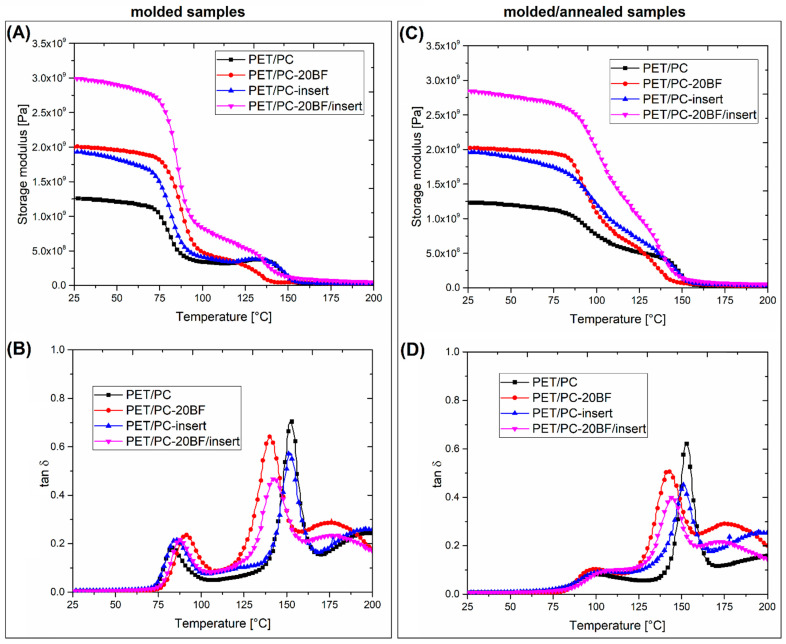
The viscoelastic properties of the PET/PC-based materials: (**A**,**B**) storage modulus and tan δ for molded samples; (**C**,**D**) related results for materials subjected to annealing.

**Figure 10:**
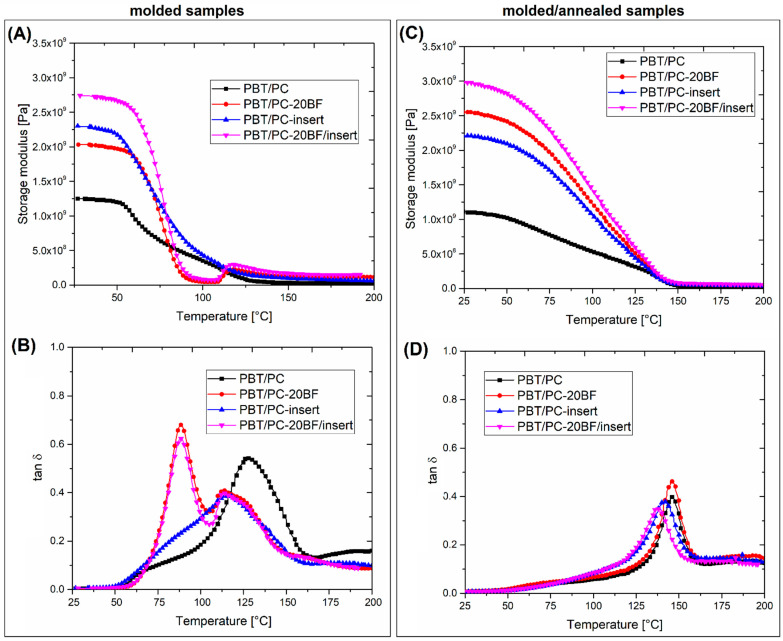
The viscoelastic properties of the PBT/PC-based materials: (**A**,**B**) storage modulus and tan δ for molded samples; (**C**,**D**) related results for materials subjected to annealing.

The thermomechanical properties of the prepared composites were analyzed using two methods: DMTA analysis and Vicat/HDT measurements. The results in the form of storage modulus and tan δ thermograms are presented in Figure 8, Figure 9 and Figure 10. The DMTA analysis allows for the analysis of the sample stiffness, which can be determined with the use of the storage modulus plots, while the phase transitions of the material can be determined using the tan δ plots analysis. The additional plots posted in Figure S2 (Supplementary Information Section) present the viscoelastic properties of the reinforcing prepregs.

The compilation in Figure 8 compares the DMTA plots of the reference pure polymer and the two polymer blends. The storage modulus plots reveal a significant difference in the heat resistance of pure polymers. This can be deduced by comparing the temperatures at which the storage modulus is significantly reduced. For the PC sample, a decreasing trend was observed around 140 °C, while the tan δ peak indicating the glass transition temperature (T_g_) was detected at 155 °C. Much lower temperature values are observed for both polyester varieties. For the PET sample, a stiffness drop was observed at 70 °C, while the T_g_ was 85 °C. The same characteristics for PBT are respectively 40 °C and 60 °C. However, it is worth remembering that for both varieties of polyesters, the decrease in stiffness associated with the T_g_ is not always directly related to the HDT or Vicat test temperature. In particular, for the PBT resin, whose crystallinity is usually relatively high, T_g_ values are not a decisive factor in determining heat resistance. In turn, for amorphous polymers like PC, the T_g_ values are closely related to the results of industrial thermomechanical tests, such as HDT. The viscoelastic properties of the PET/PC blends did not indicate miscibility between the polymers. It is confirmed by the presence of double tan δ peaks and a double-stage decrease of the blend stiffness observed on the storage modulus plot. The T_g_ peak of the PET component was at the same position, while for the PC phase, the peak was shifted from 155 °C to 150 °C. This behavior confirmed the double phase structure of the PET/PC blends and the absence of macromolecular structure miscibility. Different properties were observed in PBT/PC samples, confirming at least the partial miscibility of the blend. The appearance of the storage modulus plot suggests a single-step phase transition, since the stiffness was reduced in a linear way from around 60 °C to 125 °C. The tan δ plots also suggest a single T_g_ peak, close to 120 °C. The magnification of the tan δ thermogram reveals the presence of a slight plot shoulder at around 70 °C, which suggests a phase separation. Taking into account this type of viscoelastic behavior, it can be concluded that the PBT/PC blend is at least partly miscible.

The DMTA analysis for PET/PC-based materials is presented in Figure 9. The storage modulus plots for PET/PC-based samples reveal the two-step nature of the stiffness decrease for all samples. As can be expected, the stiffness for the PET/PC-BF/insert sample was the highest, since the prepreg structure and short BF fibers were giving the same high stiffness in the tensile test measurements. The storage modulus plots analysis reveals that the stiffness for the short fiber-reinforced sample, PET/PC/BF20, and the insert-reinforced blend was comparable, which suggests that the reinforcing efficiency of these two types of manufacturing strategies is slightly similar. The highest stiffness was recorded for the combined short fiber/insert reinforcement, which was easy to predict. Since the prepared samples were subjected to annealing, the storage modulus values were strongly influenced by the increased level of PET phase crystallinity. The most evident changes were recorded above the glass transition region, where the stiffness of all specimens was strongly improved. Compared to untreated PET/PC samples, where the storage modulus values are strongly reduced close to the glass transition region, the same reduction for annealed specimens is less sharp. Relatively high modulus values are retained up to a temperature of approximately 130 °C, where the tested materials fall within the glass transition range of the PC phase. Due to the completely amorphous nature of PC, the annealing procedure does not have a significant effect on the increase in the thermal stability of this polymer; therefore, at around 150 °C, the structural changes cause the modulus value to drop to below 100 MPa, which consequently causes the final softening of the samples. Interestingly, the performed heat treatment is also revealed during the analysis of the tan δ plots. For “molded” samples, the presence of two distinct peaks confirms the presence of two separated glass transition regions for the PET and PC phases. The heat treatment is leading to a visible reduction of the PET phase peak area, which can be correlated with a reduction in the content of the amorphous phase resulting from the growth of lamellar structures. Additionally, the peak maximum position was shifted from approximately 80 °C to 95 °C, which is associated with a decrease in the mobility of macromolecules. This phenomenon results from the increased physical interaction between the crystalline and amorphous structures, which consequently leads to the increased stiffness of the system [84,85].

The analysis of the PBT/PC-based blends revealed some important differences when compared to PET/PC materials (see Figure 10). For PBT/PC blends, the use of 20% of BF reinforcement seems to be less effective than the insert overmolding, since the initial values of the storage modulus are slightly higher for the PBT/PC-(insert) sample. The highest stiffness for the molded specimens was recorded for the PBT/PC-(BF20/insert) sample, which is the expected result since the material was prepared with short and long BF reinforcements. Interestingly, there is a visible difference between the plot appearance for unmodified PBT/PC and the short BF modified materials. Where, for samples without the dispersed short fiber reinforcement the storage modulus plots are characterized by a relatively mild decrease in value after exceeding the T_g_ value for PBT, yet for the materials with the addition of BF the stiffness changes in the glass transition region are very clear and resemble those observed for systems based on PET/PC. The most interesting observations include tan δ graph analysis, where the results for unfilled blend samples and those with 20% BF addition differ significantly. For both the PBT/PC and PBT/PC-(insert) samples, the single tan δ peak was observed, which confirmed the previous conclusion regarding the miscibility of this kind of blend. The observation of the tan δ plot for the PBT/PC-insert sample is more difficult, since the plot is flattened due to the high level of structure reinforcement. However, a single tan δ peak suggests the presence of miscibility of PC and PBT phases. Interestingly, the peak position is shifted to 115 °C, which is visibly lower than the unreinforced PBT/PC blend. It is not clear what the reason was for that type of behavior, since the only difference between these two samples is related to the presence of a composite insert. However, considering the presence of the slight curvature of the specimen after the overmolding process, it is possible that the magnitude of internal stresses is high, which might additionally influence the tan δ peak position.

For all short BF-reinforced samples, the single plot splits into two signals, suggesting the presence of two separate phases. For both samples, the PBT phase peak is located close to the temperature of 90 °C, which is largely higher than the 60 °C for the reference PBT sample. The second peak associated with the PC phase was also shifted from the initial 155 °C (for pure PC) to around 120 °C for both PBT/PC/BF20 types of samples. This behavior is very different from that observed for unmodified blends. The change in the structure of the PBT/PC system from miscible to two-phase is obviously dependent on the presence of BF fibers. However, from the perspective of the conducted research, it can only be concluded that the presence of fibers may accelerate the nucleation of the PBT crystalline phase, thereby increasing the share of the RAF phase (rigid amorphous fraction). An interesting observation is also the high value of the tan δ peak and its dominant character, suggesting an increase in the content of the amorphous phase of PBT. The conducted analysis does not allow a clear determination of the mechanism underlying these changes, prompting further research in this area. It is clear that an additional series of blends with different PBT/PC correlations would be helpful for a deeper study of the observed phenomenon.

Similar to PET/PC-based materials, the annealing procedure performed for the PBT/PC materials has an even greater impact on the viscoelastic characteristics of the tested specimens. The storage modulus values presented in Figure 10C are strongly improved compared to molded samples. Additionally, the previously observed difference in plot appearance was visibly changed, since for all plots the decrease in the modulus value is almost linear, and the differences in the values are directly related to the BF fiber content, where the highest stiffness is associated with the use of the BF20/insert system. The recorded behavior is strongly associated with the improvement in the PBT phase crystallinity, which can be confirmed in the tan δ plots (see Figure 10D). In the glass transition region of the PBT component, the tan δ peak has been reduced to a slight plot inflection, which clearly indicates the disappearance of the free volume of the amorphous PBT phase. This type of behavior is a natural consequence of the material annealing process and is commonly observed for thermoplastic polyesters [84]. The changes observed in the glass transition of the PC component are much more interesting in nature. In the case of samples not subjected to heat treatment, the position of the T_g_ peak for the PC phase was significantly shifted compared to the value for pure PC (≈155 °C). In the most extreme cases, the peak shift reached 115 °C, which can be easily related to the miscibility of the tested PBT-PC phase. Due to the annealing procedure, conducted at 140 °C, the position of the T_g_ peak of the PC phase moved to the range of 135–145 °C, a shift which was recorded for all tested samples. The observations made indicate significant changes in the structural characteristics of materials, where, in the case of revealed phenomena, only certain assumptions can be made. It is certainly an undeniable fact that the increased content of the PBT crystalline phase causes a significant fraction of PBT macromolecules to be trapped in lamellar structures. In this case, in the case of the initially miscible PBT/PC system, two separate phases are distinguished where the PC macromolecules are trapped in the interlamellar space of the PBT crystalline structures. In summary, it is clear that from a structural point of view, the observed changes in viscoelastic behavior are very complex for both PBT- and PET-based materials; however, from the point of view of the presented application, in both cases the annealing procedure seems to be an effective methodology for heat resistance enhancement.

**Figure 11:**
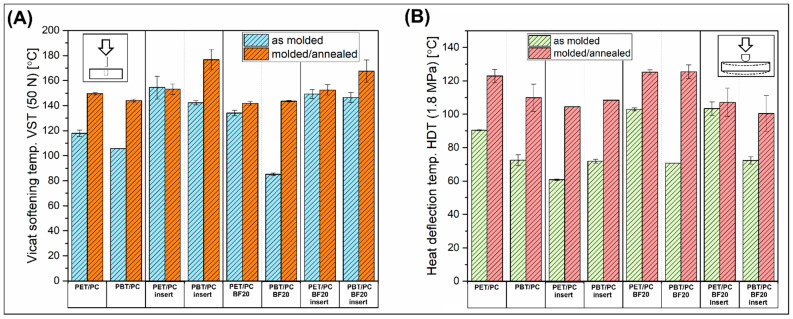
The heat resistance measurements results: (**A**) Vicat softening temperature (VST); (**B**) heat deflection temperature (HDT). Plots are collecting the results for molded and annealed samples.

The analyzed viscoelasticity can be considered as a detailed description of the resulting changes in the stiffness and damping of the material in a temperature scale, but in practice, the key application parameters are determined in the Vicat and HDT tests. The plots presented in Figure 11A present the Vicat softening temperature results for molded and annealed samples. The comparison of the VST for PET/PC and PBT/PC materials revealed that, for most of the samples, the heat treatment leads to a visible improvement in thermomechanical performance. Starting from the unreinforced blends, the VST was improved from 120 °C to 150 °C for PET/PC and from 105 °C to 135 °C for the PBT/PC sample, where the obvious reason for that was the structure crystallization. Interestingly, for the blends reinforced with composite insert, the VST for molded samples was strongly improved, reaching at least 140 °C. The results for the insert-reinforced samples are an unusual example where the measurement was performed on the reinforced surface, so the Vicat test result is indirectly the effect of the softening of the thin prepreg (1 mm) and the overmolding material located under the composite layer. The recorded VST result confirms the high reinforcing efficiency of the composite insert. Unfortunately, due to the small thickness, tests for prepregs were not performed, but it can be safely stated that the value of both the VST and HDT indexes is the highest for these materials. Interestingly, the annealing leads to VST improvement only for PBT/PC-insert samples (≈176 °C), while the VST for PET/PC-insert samples remained at the same level. It is difficult to determine the cause of this behavior. Due to the non-uniform nature of the tested samples, it may be related to slight differences in the internal stresses of the products or local differences in crystallinity below the overmolded prepreg surface. The next set of samples included materials with the addition of short BF filler, where a clear reduction in the VST value for the PBT/PC-BF20 material can be seen (≈80 °C). This result indirectly reflects the DMTA analysis, where for the discussed samples a rapid change in stiffness in the T_g_ region was also recorded. In the subsection on the DSC analysis, this phenomenon is discussed in more detail and correlated with the decrease in the crystallinity of the PBT phase. The last set of samples is represented by the materials reinforced by the double (BF20/insert) system. The presence of a large amount of basalt fibers causes high reinforcing efficiency, even at 150 °C for overmolded samples. The annealing is slightly improving these results, up to 165 °C for PBT/PC-BF20/insert sample; however, considering the VST improvement recorded for the rest of the specimens, the heat treatment was less efficient than for materials molded using the standard injection molding method.

The results of the heat deflection measurements (HDT) revealed the more favorable influence of the annealing procedure (see Figure 11B). Starting from the unmodified blends samples, the initial HDT values of 90 °C and 72 °C, respectively, for the PET/PC and PBT/PC samples, were improved to 123 °C and 110 °C. Similar behavior was observed for insert-reinforced material; however, in this case, the recorded HDT values were slightly lower, despite the introduction of basalt reinforcement. Interestingly, very similar values were observed for samples with a double reinforcement system. This case requires further discussion, as the observed phenomena are caused by sample inverse deformation, where the bending curvature is observed in the direction of the load, and, therefore, it is not the deformation value resulting from the action of the device’s weight. This untypical behavior is caused by the different stress states of these heterogeneous specimens. During heating, stress relaxation causes slight buckling of the samples, which in turn exceeds the values recorded during the HDT test, leading to relatively low measurement results. The annealing process improves the results, but the HDT value is still lower than for the corresponding PET/PC and PBT/PC samples. In this case, premature buckling was still recorded during the measurement. However, considering that thermal treatment in the T_g_ region of the PC should have removed any residual stresses, the most likely phenomenon seems to be the difference in thermal expansion between the prepreg and the overmolded part, where, due to the higher content and orientation of BF fibers, the samples still bulged. The highest HDT results were observed for PET/PC and PBT/PC materials after annealing, where both types of samples reached around 125 °C of HDT. For this type of sample, it is also important that the heat treatment was improved significantly, from the reference 103 °C and 79 °C, respectively, for PET/PC and PBT/PC.

The result of the HDT test revealed some interesting behavior associated with the presence of internal stress, a phenomenon which can be a subject of deflection simulation/validation studies. Unfortunately, many previous studies regarding this type of validation experiments revealed that the FEA models are not helpful during the modeling of such complex systems [86,87,88,89,90]. These types of experiments will require modeling changes in mechanical properties and the associated stress field changes, where these would depend on the thermal conditions during the cooling of the two-layer material. In this case, stress fluctuations would depend on thermal factors in both the overmolding material and the composite prepreg. Hence, we conclude that a simulation of this type, given the current state of knowledge, would be imprecise; however, due to the complex nature of this issue, an attempt to develop an effective technique for simulating this type of phenomena would be a major step in the development of numerical methods for simulating polymer processing processes.

### 3.3. Thermal Properties—Differential Scanning Calorimetry (DSC) Analysis

**Figure 12:**
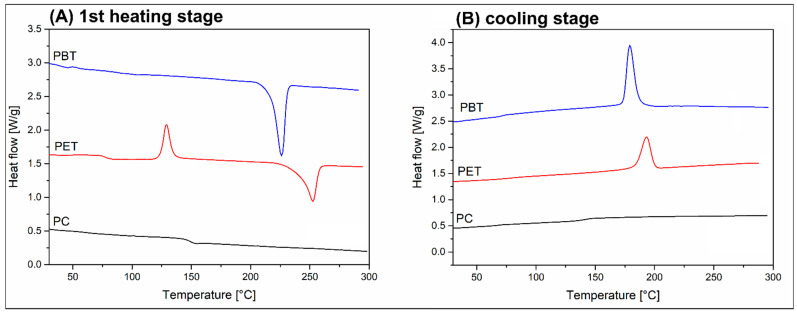
The differential scanning calorimetry (DSC) analysis results for the reference PC, PET and PBT materials: (**A**) the first heating stage plots; (**B**) cooling stage thermograms.

**Figure 13:**
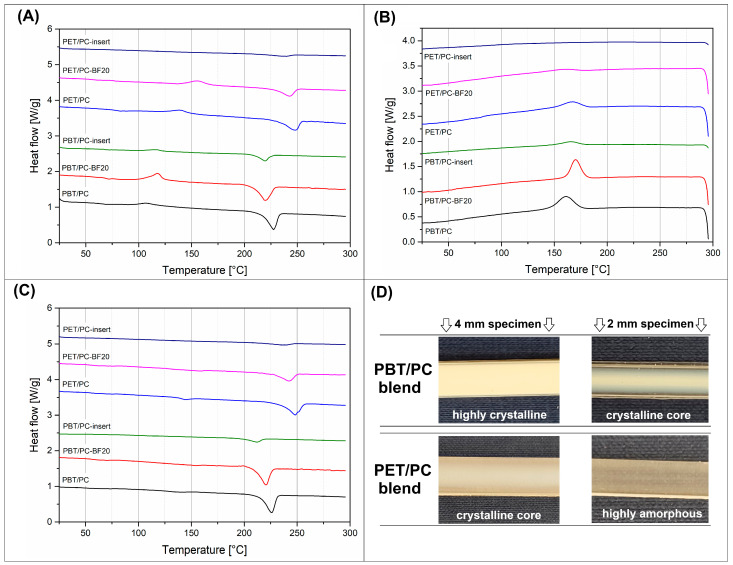
The differential scanning calorimetry (DSC) analysis results for PET/PC and PBT/PC materials: (**A**) the first heating stage plots; (**B**) cooling stage thermograms; (**C**) first heating plots of the annealed samples, (**D**) sample appearance, for specimens with varied thickness.

The thermal properties of the prepared materials were investigated using standardized heating/cooling DSC measurements. The thermograms for the reference polymers are presented in Figure 12, while the results for PET/PC and PBT/PC blends are collected in Figure 13. Additionally, the basic data and calculations obtained from the thermogram analysis are presented in Table 2. Since both types of polyesters (PET, PBT) are semi-crystalline polymers, the crystallinity was calculated.

The first heating thermograms presented in Figure 12A reveal that the addition of PC into the PET and PBT has a visible effect on the thermal properties of the blends. It is worth noting that the sample appearance was already revealing some important differences between PET- and PBT-based materials (see Figure 13D), since for the standard 4 mm specimen the PBT/PC blends were opaque in color, but after reducing the sample thickness to 2 mm only the surface layer was transparent, while the internal volume was still retained as white and non-transparent. For PET/PC materials, the 4 mm samples were characterized by a thick outer transparent layer, while the crystallized core occurred in the inner part of the sample cross-section. In contrast, the 2 mm samples were, in fact, highly transparent. The observed differences indicate a much higher crystallinity of PBT-based materials, which does not exclude the possibility of achieving a high content of the PET crystalline phase in the case of favorable thermal conditions, such as in the case of annealing or injection molding at high mold temperature.

The visible thermogram differences occurred during the melting and crystallization of the PET/PBT structure. For the reference results, pure polymers are collected in Figure 13A, while the data calculated from the DSC thermograms are listed in Table 2. The most visible changes are observed during the phase transition of the changes in the PET resin. The initial melting peak of the pure PET sample (253 °C) was shifted to 247 °C for the PET/PC blend and to 242 °C for the PET/PC/BF20 sample. This behavior suggests that the properties of the PET crystalline structure have been slightly modified. The presence of the PC component is leading to limited macromolecule mobility, which is disturbing the formation of PET lamellar structures. The reduced crystallite size is reflected in a decrease in the melting peak temperature; additionally, the cooling scans reveal slower crystallization kinetics for the PET/PC materials. Interestingly, the calculated crystallinity level of the PET phase in the PET/PC blend is similar to the results obtained for pure PET (≈23%), which again confirms that the macromolecular structure of this type of polymer blend is immiscible. The addition of the BF filler did not influence the appearance of the first heating plots; however, the crystallinity level was visibly reduced to 11%, which can be related to the increased thermal conductivity of the BF loaded material. Similar trends were recorded for PBT/PC-based materials, where the PBT phase crystallinity in the PBT/PC blend was reduced from around 28% to 21% when introducing BF filler. Our explanation of that phenomenon was mostly based on the previous studies regarding the influence of basalt filler content on the thermal conductivity [91,92]. The lack of high efficiency in nucleation of the crystalline phase of polyesters has already been confirmed for BF fibers in many publications, the results presented in the present study were not surprising [93,94]. Hence our conclusions regarding the limited growth kinetics of the crystalline phase of PET and PBT, a behavior which can be associated with both the phenomenon of improved thermal conductivity and the lack of BF nucleation interactions.

For comparative purposes, the presented charts present the DSC plots for prepreg samples; however, due to the large amount of BF reinforcement (70%), the recorded heat flow signal is very small. This makes the quantitative analysis of the melting enthalpy values very difficult and, therefore, burdened with significant error, which is why we did not include data on measurements for pressed prepregs in the table. Interestingly, even for the composite prepreg structure, the cold crystallization peak (T_cc_) was recorded, despite the fact that the cooling stem of the hydraulic press was passive, without the cooling medium, as in injection molding.

The first heating thermograms for the annealed materials revealed the presence of a highly crystalline structure for both PET/PC and PBT/PC types of materials. The exothermic peak correlated with the cold crystallization phenomenon is no longer observed for any of the samples. The crystallinity for the PET/PC and the PET/PC-BF20 samples was 41% and 34%, respectively. The PBT-based materials reached 45% and 56.5%, respectively, for the pure blend and the BF BF-loaded sample. The increase in crystallinity is therefore significant, especially for materials based on the PET/PC system.

## 4. Conclusions

The results presented in the article indicate the possibility of obtaining a durable connection of composite prepregs and overmolded material in both tested cases. In the case of the PET/PC material, the adhesion at the connection interface was visibly stronger, which is probably related to differences in the morphology of the tested systems. The probable factors limiting diffusion phenomena during overmolding of PBT/PC prepregs include the partial miscibility of this system, which reduces the mobility of the amorphous phase at the interface of the joined materials. Another potential factor is the high kinetics of PBT crystalline phase formation, which limits the time to diffuse between the phase boundaries. The results indicate that the introduction of short BF filler into the polymer matrix did not adversely affect the strength of the connection with the prepreg, but it had a significant impact on the improvement of mechanical properties. The combined reinforcement system using short fibers and prepreg allowed for obtaining over 8 GPa of E modulus and over 160 MPa of tensile strength; importantly, the impact strength of the tested materials also exceeded 30 kJ/m^2^.

The conducted tests demonstrate high potential for the application of the developed composites. However, the research identified several interesting areas that require further testing. First, technological trials should be conducted at elevated injection mold temperatures, thereby eliminating the annealing step for the finished components. In the discussed studies, thermal treatment was aimed at testing how increasing the crystalline phase would affect thermomechanical parameters. However, considering the efficiency of actual technological processes, additional treatment is economically unfavorable. A more beneficial strategy refers to the implementation of the overmolding procedure at high mold temperature; however, at this stage of the research, any attempt to calculate cycle time or costs would be misleading. It is worth noting that for an industrial cycle, where the thickness of the overmolded part would be significantly smaller than that of ISO standard samples (4 mm), the cooling cycle time associated with reducing the thickness of the plastic mass could be reduced. Therefore, many process scenarios can be identified, where the fundamental process and potential changes in time and costs will be primarily influenced by the shape and size of the product. However, the repeatability, efficiency and unit price of any products manufactured using overmolded techniques are certainly more favorable compared to the processing techniques for thermoset-based laminates.

Another interesting aspect with potential for further research is the characterization of the polymer blends themselves, particularly the PBT/PC system, which demonstrates miscibility. From the perspective of describing phase transitions, further detailed studies of the interactions between the amorphous and crystalline phases of PBT in this system seem necessary.

## Figures and Tables

**Table 1:** The list of sample markings and material formulations.

	PC [%]	PET [%]	PBT [%]	Short Basalt Fiber (BF) [%]	Composite Prepreg (Insert) [-/1]
Standard injection molding					
PC	100	-	-	-	-
PET	-	100	-	-	-
PBT	-	-	100	-	-
PET/PC(50:50)	50	50	-	-	-
PET/PC-BF20	40	40	0	20	-
PBT/PC(50:50)	50	-	50	-	-
PBT/PC-BF20	40	-	40	20	-
Overmolding process					
PET/PC-insert	50	50	-	-	1
PET/PC-BF20/insert	40	40	0	20	1
PBT/PC-insert	50	-	50	-	1
PBT/PC-BF20/insert	40	-	40	20	1

**Table 2:** The data collected from the DSC thermograms.

	$∆H_{cc}$ [J/g]	$∆H_{m}$ [J/g]	${peak}_{m}$ [°C]	${peak}_{CC}$ [°C]	${peak}_{C}$ [°C]	$X_{c}$ [°C]
Reference						
PET	17.0	48.9	253.0	128.9	193.7	22.8
PBT	- *	51.9	227.1	-	178.3	36.5
Molded samples						
PET/PC(50/50)	9.1	24.1	246.9	138.2	167.8	21.3
PET/PC-BF20	12.4	18.6	242.3	154.9	162.0	11.1
PBT/PC(50/50)	9.7	29.3	225.9	-	164.5	27.6
PBT/PC-BF20	21.0	32.7	219.9	118.0	170.1	20.7
Samples after annealing						
PET/PC(50/50)	-	28.9	248.0	-	165.8	41.2
PET/PC-BF20	-	18.9	242.3	-	161.1	33.8
PBT/PC(50/50)	-	31.9	225.9	-	164.5	44.9
PBT/PC-BF20	-	32.1	220.8	-	172.0	56.5

* value not detected (-).

## Data Availability

The original contributions presented in this study are included in the article. Further inquiries can be directed to the corresponding author.

## Supplementary Materials

The following supporting information can be downloaded at: https://www.mdpi.com/article/10.3390/polym18010054/s1, Figure S1: The comparison of the tensile test samples after fracture; Figure S2: The viscoelastic properties of the reference samples and prepreg materials: (A) storage modulus and (B) tan δ plots; Table S1: The list of mechanical properties obtained during the static tensile/flexural measurements and Izod impact tests.

## References

[1] Muniyan V., Vijay Kumar V., Suyambulingam I., Priyadharshini S., Divakaran D., Rangappa S.M., Siengchin S. (2025). A Review of Recent Advancements in the Impact Response of Fiber Metal Laminates. Heliyon.

[2] Trzepieciński T., Najm S.M., Sbayti M., Belhadjsalah H., Szpunar M., Lemu H.G. (2021). New Advances and Future Possibilities in Forming Technology of Hybrid Metal–Polymer Composites Used in Aerospace Applications. J. Compos. Sci..

[3] Zhang J., Lin G., Vaidya U., Wang H. (2023). Past, Present and Future Prospective of Global Carbon Fibre Composite Developments and Applications. Compos. Part B Eng..

[4] Landi D. (2024). A New Interactive Design Method for Carbon Fibres Laminate Component. Int. J. Interact. Des. Manuf..

[5] Tayebi S.T., Sambucci M., Valente M. (2024). Waste Management of Wind Turbine Blades: A Comprehensive Review on Available Recycling Technologies with A Focus on Overcoming Potential Environmental Hazards Caused by Microplastic Production. Sustainability.

[6] Alavi Z., Khalilpour K., Florin N., Hadigheh A., Hoadley A. (2025). End-of-Life Wind Turbine Blade Management across Energy Transition: A Life Cycle Analysis. Resour. Conserv. Recycl..

[7] Lan T., Wang B., Zhang J., Wei H., Liu X. (2023). Utilization of Waste Wind Turbine Blades in Performance Improvement of Asphalt Mixture. Front. Mater.

[8] Aldosari S.M., AlOtaibi B.M., Alblalaihid K.S., Aldoihi S.A., AlOgab K.A., Alsaleh S.S., Alshamary D.O., Alanazi T.H., Aldrees S.D., Alshammari B.A. (2024). Mechanical Recycling of Carbon Fiber-Reinforced Polymer in a Circular Economy. Polymers.

[9] Ballout W., Sallem-Idrissi N., Sclavons M., Doneux C., Bailly C., Pardoen T., Van Velthem P. (2022). High Performance Recycled CFRP Composites Based on Reused Carbon Fabrics through Sustainable Mild Solvolysis Route. Sci. Rep..

[10] Giorgini L., Benelli T., Brancolini G., Mazzocchetti L. (2020). Recycling of Carbon Fiber Reinforced Composite Waste to Close Their Life Cycle in a Cradle-to-Cradle Approach. Curr. Opin. Green Sustain. Chem..

[11] Szatkowski P., Twaróg R. (2024). Thermal Recycling Process of Carbon Fibers from Composite Scrap—Characterization of Pyrolysis Conditions and Determination of the Quality of Recovered Fibers. Fibers.

[12] Ren Y., Xu L., Shang X., Shen Z., Fu R., Li W., Guo L. (2022). Evaluation of Mechanical Properties and Pyrolysis Products of Carbon Fibers Recycled by Microwave Pyrolysis. ACS Omega.

[13] Li X., Xu L., Ren Y., Nan Z., Xiao S., Shen Z. (2024). Research on Microwave Pyrolysis Recovery and Reuse Performance of Carbon Fiber Composites. Polymers.

[14] Hubbard A.M., Ren Y., Papaioannou P., Sarvestani A., Picu C.R., Konkolewicz D., Roy A.K., Varshney V., Nepal D. (2022). Vitrimer Composites: Understanding the Role of Filler in Vitrimer Applicability. ACS Appl. Polym. Mater..

[15] Zheng J., Png Z.M., Ng S.H., Tham G.X., Ye E., Goh S.S., Loh X.J., Li Z. (2021). Vitrimers: Current Research Trends and Their Emerging Applications. Mater. Today.

[16] Ye G., Wang C., Guo Y., Yang Q., Huo S. (2025). Vitrimer as a Sustainable Alternative to Traditional Thermoset: Recent Progress and Future Prospective. ACS Polym. Au.

[17] Denissen W., Winne J.M., Du Prez F.E. (2016). Vitrimers: Permanent Organic Networks with Glass-like Fluidity. Chem. Sci..

[18] Sun X., Belnoue J.P.H., Wang W.T., Kim B.C., Hallett S.R. (2021). “Un-Forming” Fibre-Steered Preforms: Towards Fast and Reliable Production of Complex Composites Parts. Compos. Sci. Technol..

[19] Gómez C., Tobalina-Baldeon D., Cavas F., Sanz-Adan F. (2022). Geometrical Optimization of Thermoforming Continuous Fibers Reinforced Thermoplastics with Finite Element Models: A Case Study. Compos. Part B Eng..

[20] Wakeman M.D., Rudd C.D. (2000). Compression Molding of Thermoplastic Composites. Comprehensive Composite Materials.

[21] Duhovic M., Bhattacharyya D. (2011). Knitted Fabric Composites. Advances in Knitting Technology.

[22] Tan Y., Wang X., Wu D. (2015). Preparation, Microstructures, and Properties of Long-Glass-Fiber-Reinforced Thermoplastic Composites Based on Polycarbonate/Poly (Butylene Terephthalate) Alloys. J. Reinf. Plast. Compos..

[23] Zhang D., He M., Qin S., Yu J. (2017). Effect of Fiber Length and Dispersion on Properties of Long Glass Fiber Reinforced Thermoplastic Composites Based on Poly(Butylene Terephthalate). RSC Adv..

[24] Bazan P., Kuciel S., Sądej M. (2020). The Influence of Adding Long Basalt Fiber on the Mechanical and Thermal Properties of Composites Based on Poly (Oxymethylene). J. Thermoplast. Compos. Mater..

[25] Bartus S.D., Vaidya U.K. (2005). Performance of Long Fiber Reinforced Thermoplastics Subjected to Transverse Intermediate Velocity Blunt Object Impact. Compos. Struct..

[26] Alwekar S., Ogle R., Kim S., Vaidya U. (2021). Manufacturing and Characterization of Continuous Fiber-Reinforced Thermoplastic Tape Overmolded Long Fiber Thermoplastic. Compos. Part B Eng..

[27] Zhang J.M., Reynolds C.T., Peijs T. (2009). All-Poly(Ethylene Terephthalate) Composites by Film Stacking of Oriented Tapes. Compos. Part A Appl. Sci. Manuf..

[28] Ary Subagia I.D.G., Kim Y., Tijing L.D., Kim C.S., Shon H.K. (2014). Effect of Stacking Sequence on the Flexural Properties of Hybrid Composites Reinforced with Carbon and Basalt Fibers. Compos. Part B Eng..

[29] Wang J., Chen J., Dai P., Wang S., Chen D. (2015). Properties of Polypropylene Single-Polymer Composites Produced by the Undercooling Melt Film Stacking Method. Compos. Sci. Technol..

[30] Liu J., Quan D., Ma Y., Bai S., Wang X., Yang X., Zhao G. (2025). An Integrated Stamp Forming and Injection Overmolding Process for Thermoplastic Composite Components: Enhancing Overmolding Interface. J. Manuf. Process..

[31] Akkerman R., Bouwman M., Wijskamp S. (2020). Analysis of the Thermoplastic Composite Overmolding Process: Interface Strength. Front. Mater.

[32] Boros R., Rajamani P.K., Kovács J.G. (2018). Thermoplastic Overmolding onto Injection-Molded and in Situ Polymerization-Based Polyamides. Materials.

[33] Jerpdal L., Schuette P., Ståhlberg D., åkermo M. (2020). Influence of Temperature during Overmolding on the Tensile Modulus of Self-Reinforced Poly (Ethylene Terephthalate) Insert. J. Appl. Polym. Sci..

[34] Andrzejewski J., Szostak M. (2020). Preparation of Hybrid Poly(Lactic Acid)/Flax Composites by the Insert Overmolding Process: Evaluation of Mechanical Performance and Thermomechanical Properties. J. Appl. Polym. Sci..

[35] Bula K., Korzeniewski B. (2022). Polyamide 6-Aluminum Assembly Enhanced by Laser Microstructuring. Polymers.

[36] Vasconcelos R.L., Oliveira G.H.M., Amancio-Filho S.T., Canto L.B. (2023). Injection Overmolding of Polymer-Metal Hybrid Structures: A Review. Polym. Eng. Sci..

[37] Geminger T., Jarka S. (2016). Injection Molding of Multimaterial Systems. Specialized Injection Molding Techniques.

[38] Novák L., Fojtl L., Kadlečková M., Maňas L., Smolková I., Musilová L., Minařík A., Mráček A., Sedláček T., Smolka P. (2021). Surface Modification of Metallic Inserts for Enhancing Adhesion at the Metal–Polymer Interface. Polymers.

[39] Paramasivam A. (2025). Investigating the Failure Behavior of Over-Molded Thermoplastic Composites: Experimental Testing and Numerical Modelling. Appl. Compos. Mater..

[40] Héri-Szuchács A., Kovács J.G. (2024). Calculation of the Bonding Strength of Semi-Crystalline Polymers during Overmolding. Polym. Test..

[41] Reyes E., Tardif X., Bailleul J.L., Allanic N., Sobotka V. (2022). Inverse Heat Transfer Optimization of Stamping with Over-Molding Process Involving High Performance Thermoplastic Composites: Experimental Validation. Int. J. Mater. Form..

[42] Andrzejewski J., Przyszczypkowski P., Szostak M. (2018). Development and Characterization of Poly(Ethylene Terephthalate) Based Injection Molded Self-Reinforced Composites. Direct Reinforcement by Overmolding the Composite Inserts. Mater. Des..

[43] Lombardo B.S., Keskkula H., Paul D.R. (1994). Influence of ABS Type on Morphology and Mechanical Properties of PC/ABS Blends. J. Appl. Polym. Sci..

[44] Nishino K., Shindo Y., Takayama T., Ito H. (2017). Improvement of Impact Strength and Hydrolytic Stability of PC/ABS Blend Using Reactive Polymer. J. Appl. Polym. Sci..

[45] Chiu H.T., Huang J.K., Kuo M.T., Huang J.H. (2018). Characterisation of PC/ABS Blend during 20 Reprocessing Cycles and Subsequent Functionality Recovery by Virgin Additives. J. Polym. Res..

[46] Bärwinkel S., Seidel A., Hobeika S., Hufen R., Mörl M., Altstädt V. (2016). Morphology Formation in PC/ABS Blends during Thermal Processing and the Effect of the Viscosity Ratio of Blend Partners. Materials.

[47] Andrzejewski J., Mohanty A.K., Misra M. (2020). Development of Hybrid Composites Reinforced with Biocarbon/Carbon Fiber System. The Comparative Study for PC, ABS and PC/ABS Based Materials. Compos. Part B Eng..

[48] Öge T.Ö., Öge M. (2025). Prediction of Tribological Properties of PC-PBT/GNP-MWCNT Nanocomposites Using Machine Learning Models. J. Appl. Polym. Sci..

[49] Wang K., Wu J., Zeng H. (2001). Microstructures and Fracture Behavior of Glass-Fiber Reinforced PBT/PC/E-GMA Elastomer Blends-1: Microstructures. Compos. Sci. Technol..

[50] Rezaei M.H., Bagheri R. (2026). Enhancing Adhesion and Mechanical Properties of Recycled-Poly(Ethylene Terephthalate)/Polycarbonate/Glass Fiber Blends Using Copolymer Modifiers. Polymer.

[51] Xiong Z., Sun Y., Wang L., Guo Z., Yu J. (2012). Electrical Conductivities of Carbon Nanotube-Filled Polycarbonate/Polyester Blends. Sci. China Chem..

[52] Vyavahare S.A., Kharat B.M., More A.P. (2024). Polybutylene Terephthalate (PBT) Blends and Composites: A Review. Vietnam. J. Chem..

[53] Andrzejewski J. (2023). The Use of Recycled Polymers for the Preparation of Self-Reinforced Composites by the Overmolding Technique: Materials Performance Evaluation. Sustainability.

[54] Seelig T., Giessen E. (2007). Effects of Microstructure on Crack Tip Fields and Fracture Toughness in PC/ABS Polymer Blends. Int. J. Fract..

[55] Bai L., Liu Z., Yu C., Ma M., Chen S., Shi Y., He H., Wang X. (2022). Enhanced Interfacial Adhesion for Effectively Stress Transfer Inducing the Plastic Deformation of Matrix towards High-Toughness PC/PBT/EMA-GMA Blends. Polymer.

[56] Shi P., Tang J., Duan H. (2021). Influence of Different Tougheners on the Properties of PC/PBT Alloy. J. Mater. Sci. Chem. Eng..

[57] Dal Lago E., Boaretti C., Piovesan F., Roso M., Lorenzetti A., Modesti M. (2018). The Effect of Different Compatibilizers on the Properties of a Post-Industrial PC/PET Blend. Materials.

[58] Al-Jabareen A., Illescas S., Maspoch M.L., Santana O.O. (2010). Effects of Composition and Transesterification Catalysts on the Physico-Chemical and Dynamic Properties of PC/PET Blends Rich in PC. J. Mater. Sci..

[59] Luo F., Liu X., Liu C., Ma J., Wang X., Shen C. (2019). Dynamic Viscoelasticity and Molecular Orientation in Uniaxially Drawn PC/PET Blends. J. Appl. Polym. Sci..

[60] Jerpdal L., Åkermo M. (2014). Influence of Fibre Shrinkage and Stretching on the Mechanical Properties of Self-Reinforced Poly (Ethylene Terephthalate) Composite. J. Reinf. Plast. Compos..

[61] Barczewski M., Mysiukiewicz O., Matykiewicz D., Kloziński A., Andrzejewski J., Piasecki A. (2020). Synergistic Effect of Different Basalt Fillers and Annealing on the Structure and Properties of Polylactide Composites. Polym. Test..

[62] Kong Y., Hay J.N. (2004). The Effect of Annealing on the Crystallization of Poly(Ethylene Terephthalate)/Polycarbonate Blends. J. Polym. Sci. Part B Polym. Phys..

[63] Andrzejewski J., Chmielewski P., Osiński F., Markowski M., Marciniak-Podsadna L., Piasecki A. (2024). Use of Recycled Poly(Ethylene Terephthalate)-Based (RPET) Blends in Additive Manufacturing Techniques to Prepare Sustainable, Tough, and Heat-Resistant Parts. ACS Sustain. Chem. Eng..

[64] Bhandari S., Lopez-Anido R.A., Gardner D.J. (2019). Enhancing the Interlayer Tensile Strength of 3D Printed Short Carbon Fiber Reinforced PETG and PLA Composites via Annealing. Addit. Manuf..

[65] Marchese P., Celli A., Fiorini M., Gabaldi M. (2003). Effects of Annealing on Crystallinity and Phase Behaviour of PET/PC Block Copolymers. Eur. Polym. J..

[66] (2019). Plastics—Determination of Tensile Properties.

[67] (2010). Plastics—Determination of Charpy Impact Properties.

[68] Wach R.A., Wolszczak P., Adamus-Wlodarczyk A. (2018). Enhancement of Mechanical Properties of FDM-PLA Parts via Thermal Annealing. Macromol. Mater. Eng..

[69] Orue A., Eceiza A., Arbelaiz A. (2018). The Effect of Sisal Fiber Surface Treatments, Plasticizer Addition and Annealing Process on the Crystallization and the Thermo-Mechanical Properties of Poly(Lactic Acid) Composites. Ind. Crops Prod..

[70] Simmons H., Tiwary P., Colwell J.E., Kontopoulou M. (2019). Improvements in the Crystallinity and Mechanical Properties of PLA by Nucleation and Annealing. Polym. Degrad. Stab..

[71] Ahmad Saidi M.A., Hassan A., Wahit M.U., Lai J.C. Mechanical and Thermal Properties of Polyethylene Terephthalate/Polybutylene Terephthalate Blends. Proceedings of the 7th International Graduate Conference on Engineering, Science and Humanities, IGCESH.

[72] Wasti S., Clarkson C., Johnston E., Pu Y., Bhagia S., Tekinalp H., Ozcan S., Vaidya U. (2025). Sizing of Discontinuous Natural Fibers: Effect of Sizing Approach and Sizing Concentration on Composite Properties. Compos. Part A Appl. Sci. Manuf..

[73] Kahl C., Feldmann M., Sälzer P., Heim H.P. (2018). Advanced Short Fiber Composites with Hybrid Reinforcement and Selective Fiber-Matrix-Adhesion Based on Polypropylene—Characterization of Mechanical Properties and Fiber Orientation Using High-Resolution X-Ray Tomography. Compos. Part A Appl. Sci. Manuf..

[74] Andrzejewski J., Gapiński B., Islam A., Szostak M. (2020). The Influence of the Hybridization Process on the Mechanical and Thermal Properties of Polyoxymethylene (POM) Composites with the Use of a Novel Sustainable Reinforcing System Based on Biocarbon and Basalt Fiber (BC/BF). Materials.

[75] Barczewski M., Mysiukiewicz O., Lewandowski K., Nowak D., Matykiewicz D., Andrzejewski J., Skórczewska K., Piasecki A. (2020). Effect of Basalt Powder Surface Treatments on Mechanical and Processing Properties of Polylactide-Based Composites. Materials.

[76] Andrzejewski J., Danielak A., Piasecki A., Islam A., Szostak M. (2023). Biocarbon-Based Sustainable Reinforcing System for Technical Polymers. The Structure-Properties Correlation between Polycarbonate (PC) and Polybutylene Terephthalate (PBT)-Based Blends Containing Acrylonitrile-Butadiene-Styrene (ABS). Sustain. Mater. Technol..

[77] Aniśko J., Bartczak D., Barczewski M. (2023). Limitations of Short Basalt Fibers Use as an Effective Reinforcement of Polyethylene Composites in Rotational Molding Technology. Adv. Sci. Technol. Res. J..

[78] Tábi T., Égerházi A.Z., Tamás P., Czigány T., Kovács J.G. (2014). Investigation of Injection Moulded Poly(Lactic Acid) Reinforced with Long Basalt Fibres. Compos. Part A Appl. Sci. Manuf..

[79] Chowdhury I.R., Pemberton R., Summerscales J. (2022). Developments and Industrial Applications of Basalt Fibre Reinforced Composite Materials. J. Compos. Sci..

[80] Rochardjo H.S.B., Budiyantoro C. (2021). Manufacturing and Analysis of Overmolded Hybrid Fiber Polyamide 6 Composite. Polymers.

[81] Jiang W., Chen C., Deng T., Wang X., Huang Z., Zhou H., Zhou H. (2022). Effect of Material and Processing Parameters on Fiber Pinning Effect and Resultant Interfacial Bonding Strength of CF/PEEK Bilayer Parts in Overmolding Process. Polym. Test..

[82] Yan B., Wu H., Jiang G., Guo S., Huang J. (2010). Interfacial Crystalline Structures in Injection Over-Molded Polypropylene and Bond Strength. ACS Appl. Mater. Interfaces.

[83] Giusti R., Lucchetta G. (2014). Modeling the Adhesion Bonding Mechanism in Overmolding Hybrid Structural Parts for Lightweight Applications. Key Engineering Materials.

[84] Liu J., Zhao X., Ye L. (2020). Compatibility and Toughening Mechanism of Poly(Ethylene Terephthalate)/Polycarbonate Blends. Polym. Int..

[85] Meziane O., Bensedira A.R., Guessoum M. (2021). Morphological and Thermal Characterization of an Immiscible Catalyzed Polymer Blends (PC/PET). Polym. Polym. Compos..

[86] Pozorski Z., Andrzejewski J. (2025). Experimental Determination of Mechanical Properties of 3D Printed PLA. Methodology for Testing Orthotropic Materials. Polym. Test..

[87] Hubmann M., Madadnia B., Groten J., Pletz M., Vanfleteren J., Stadlober B., Bossuyt F., Kaur J., Lucyshyn T. (2022). Process Optimization of Injection Overmolding Structural Electronics with Regard to Film Distortion. Polymers.

[88] Qiu J., Xu Z., Cai C., Chen D., Huang S., Hu C., He X. (2025). A Novel Model for Predicting Deformation of Thermoplastic Composites during Heat-Pressing Process. Compos. Part B Eng..

[89] Zhao Z., Zhang J., Bi R., Chen C., Yao J., Liu G. (2023). Study on the Overmolding Process of Carbon-Fiber-Reinforced Poly (Aryl Ether Ketone) (PAEK)/Poly (Ether Ether Ketone) (PEEK) Thermoplastic Composites. Materials.

[90] Hubmann M., Bakr M., Groten J., Pletz M., Vanfleteren J., Bossuyt F., Madadnia B., Stadlober B. (2023). Parameter Study on Force Curves of Assembled Electronic Components on Foils during Injection Overmolding Using Simulation. Micromachines.

[91] Fareez U.N.M., Loudiy A., Erkartal M., Yilmaz C. (2025). Basalt Fiber Reinforced Polymers: A Recent Approach to Electromagnetic Interference (EMI) Shielding. J. Polym. Sci..

[92] Ramesh V., Anand P. (2021). Thermal Analysis of Kevlar/Basalt Reinforced Hybrid Polymer Composite. Mater. Res. Express.

[93] Pan H., Wang X., Jia S., Lu Z., Bian J., Yang H., Han L., Zhang H. (2021). Fiber-Induced Crystallization in Polymer Composites: A Comparative Study on Poly(Lactic Acid) Composites Filled with Basalt Fiber and Fiber Powder. Int. J. Biol. Macromol..

[94] Barczewski M., Mysiukiewicz O., Andrzejewski J., Piasecki A., Strzemięcka B., Adamek G. (2021). The Inhibiting Effect of Basalt Powder on Crystallization Behavior and the Structure-Property Relationship of α-Nucleated Polypropylene Composites. Polym. Test..

