# Supplementary material for: The Use of the Overmolding Technique for the Preparation of Basalt Fiber (BF)-Based Composite, the Comparative Study of Poly(ethylene terephthalate)/Polycarbonate—PET/PC and Poly(butylene terephthalate)—PBT/PC Blends
Source: Polymers (Basel). 2025 Dec 24;18(1):54. doi: 10.3390/polym18010054 (PMC12787415; doi:10.3390/polym18010054)
Supplement: Supplementary file 1 [file polymers-18-00054-s001.zip › polymers-4057058-supplementary.pdf]

Supplementary Information Section

The use of the overmolding technique for the preparation of basalt fiber (BF)-based composite. The comparative study of poly(ethylene terephthalate)/polycarbonate - PET/PC, and poly(butylene terephthalate) - PBT/PC blends.

Jacek Andrzejewski [1]*, Wiktoria Gosławska [2], Michalina Salamaga [3], Weronika Zgoła[3] and Mateusz Barczewski [1]

[1] Institute of Material Technology, Poznan University of Technology, Piotrowo 3 Str., 61-138 Poznan, Poland, mateusz.barczewski@put.poznan.pl
[2] Faculty of Materials Engineering and Technical Physics, Poznan University of Technology, Piotrowo 3 Str., 60-965 Poznan, Poland, wiktoria.goslawska@student.put.poznan.pl (W.G.)
[3] Faculty of Mechanical Engineering, Poznan University of Technology, Piotrowo 3 Str., 60-965 Poznan, Poland, michalina.salamaga@gmail.com (M.S.), werko147@wp.pl (W.Z.)

* Correspondence: jacek.andrzejewski@put.poznan.pl, tel. 61 647 5858 (JA)

Abstract

The presented study is focused on the evaluation of the mechanical and heat resistance performance of the polyester-based injection molded components. For comparative purposes, we use poly(ethylene terephthalate)/polycarbonate blend (PET/PC) and poly(butylene terephthalate)/polycarbonate (PBT/PC) mixture, where both types of polymer blends were used as a matrix for different types of basalt fiber (BF) reinforced composites. The investigated molding procedure consists of injection overmolding of the compo-site prepreg (insert). During the technological procedure, various material configurations were used, including overmolding with both unmodified blends and a composition with additional short basalt fibers. The results confirmed that the best balance of properties was obtained for complex parts reinforced with short BF and overmolded insert, where the tensile modulus can reach 8 GPa, while the impact strength was more than 30 kJ/m2. The results of comparative tests indicate a significantly higher strength of overmolding joints for PET/PC-based materials. The relatively low heat deflection temp. (HDT) of around 70 °C after the injection molding procedure, can be successfully improved by the annealing treatment, where the HDT can reach around 120 °C. The structural tests revealed that, besides some differences in crystallinity between the PET and PBT-based blends, the thermomechanical performance of the manufactured composites is almost similar. It is worth pointing out the fundamental differences in the miscibility of the investigated blend systems, where for the PBT/PC mixture, structural tests confirm the miscibility of polymer phases, while PET/PC particles are immiscible.

Keywords: polymer blends; thermoplastic polyesters; basalt fiber, injection molding; mechanical performance; heat resistance; hybrid materials

Table S1. The list of mechanical properties obtained during the static tensile/flexural measurements, and Izod impact tests.

	Tensile test			Charpy test
samples	Modulus [MPa]	Strength [MPa]	Elongation at break [%]	Impact strength [kJ/m^2]
**Reference materials**				
PET	3243 (±121)	72.5 (±3.0)	113 (±19)	2.6 (±0.1)
PBT	2707 (±43)	55.9 (±2.9)	131 (±2.9)	5.1 (±0.4)
PC	2565 (±13)	64.1 (±0.4)	55 (±12)	10.4 (±0.4)
**PET/PC-based materials**				
PET/PC(50-50)	2 442 (±8)	61.2 (±0.8)	94.5 (±2.6)	5.2 (±0.5)
PET/PC-BF20	5 963 (±90)	103.4 (±0.5)	3.0 (±0.1)	4.9 (±0.1)
PET/PC-insert	5 434 (±302)	100.6 (±11.9)	3.6 (±0.8)	29.1 (±5.7)
PET/PC-(BF20/insert)	7 589 (±305)	157.5 (±3.6)	2.7 (±0.5)	32.7 (±4.6)
PET/PC(prepreg)	25 201 (±1 334)	366.1 (±27.9)	3.3 (±0.1)	65.8 (±19.9)
**PBT/PC-based materials**				
PBT/PC(50-50)	2512 (±12)	63.6 (±0.7)	113.1 (±18.9)	4.8 (±0.1)
PBT/PC-BF20	5730 (±81)	110.9 (±0.4)	3.4 (±0.1)	6.5 (±0.2)
PBT/PC-insert	5665 (±84)	135.0 (±20.5)	2.7 (±0.5)	21.5 (±4.6)
PBT/PC-(BF20/insert)	8349 (±123)	169.3 (±27.2)	3.7 (±0.5)	33.5 (±3.8)
PBT/PC (prepreg)	22 795 (±316)	345.7 (±9.8)	4.3 (±0.7)	43.7 (±3.9)

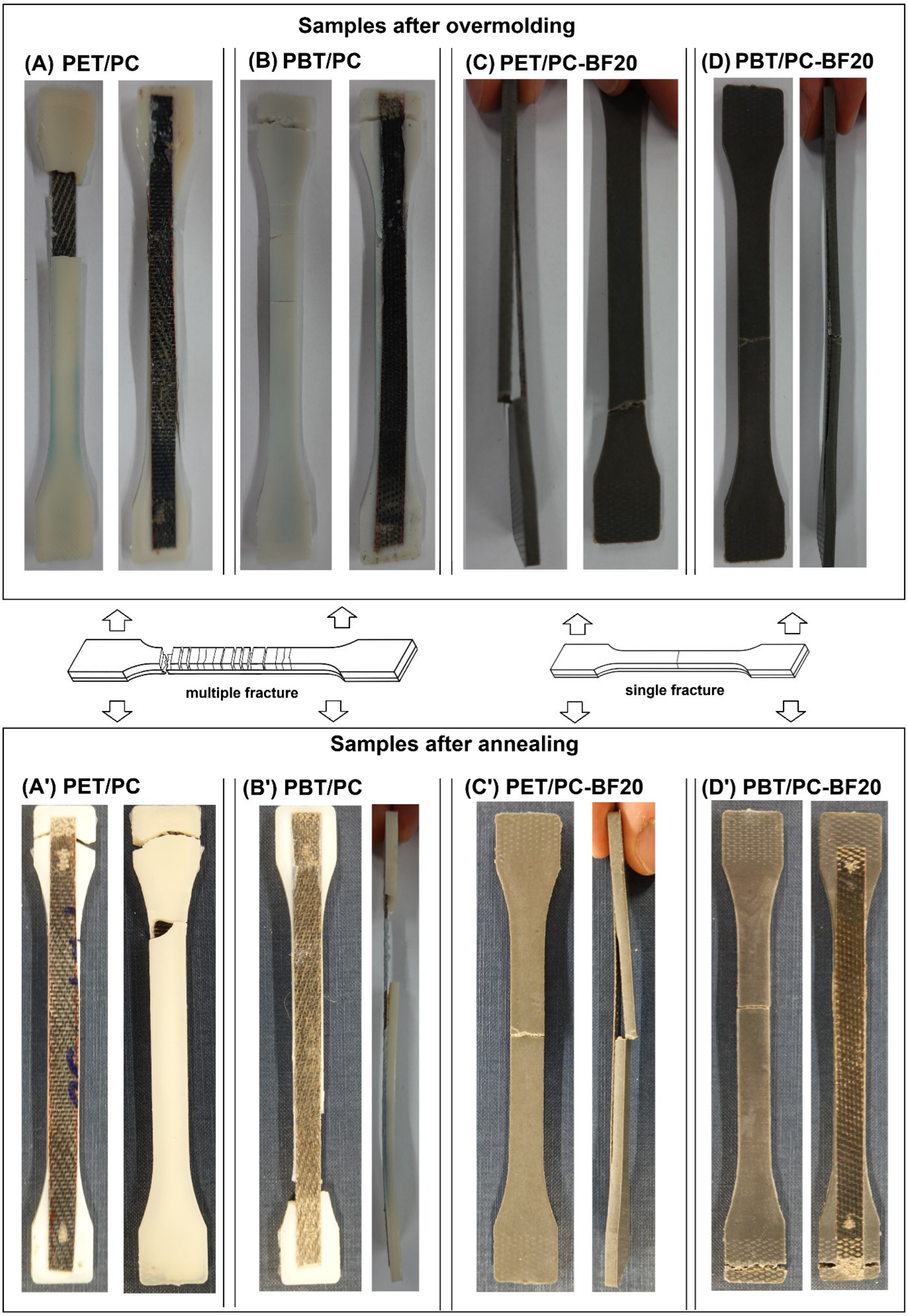

Figure S1. The comparison of the tensile test samples after fracture.

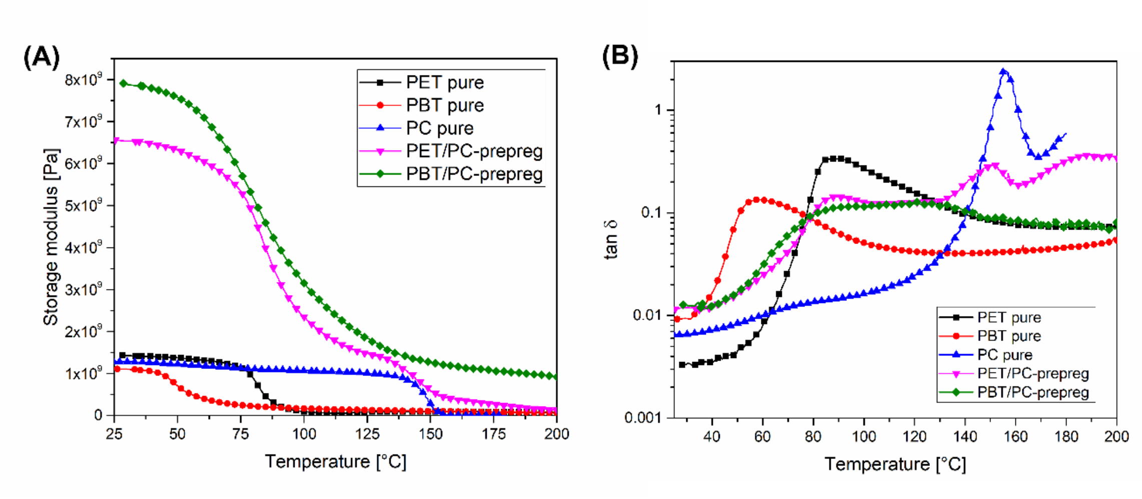

Figure S2. The viscoelastic properties of the reference samples and prepreg materials: (A) storage modulus and (B) tan δ plots.
